# Assessing the capability of the corneal blink reflex to display neurological changes following subconcussive head impacts

**DOI:** 10.3389/fneur.2025.1589577

**Published:** 2025-05-14

**Authors:** Osamudiamen S. Ogbeide, Madeleine K. Nowak, Lillian Klemsz, Dena Garner, Keisuke Kawata

**Affiliations:** ^1^Department of Kinesiology, Indiana University School of Public Health-Bloomington, Bloomington, IN, United States; ^2^Department of Psychiatry, Boston University Chobanian and Avedisian School of Medicine, Boston, MA, United States; ^3^The Translational Research Center for TBI and Stress Disorders (TRACTS) at VA Boston Healthcare System, Boston, MA, United States; ^4^Department of Health and Human Performance, The Citadel, Charleston, SC, United States; ^5^Program in Neuroscience, The College of Arts and Sciences, Indiana University, Bloomington, IN, United States

**Keywords:** traumatic brain injury, blink reflex, oculomotor, subconcussion, concussion, soccer

## Abstract

**Introduction:**

This study examines the capability of detecting neurological changes caused by subconcussive head impacts by analyzing the blink reflex of an individual when they encounter puffs of air as a stimulus.

**Methods:**

Following attrition and technical issues, 26 participants (15 females, 11 males: age ± SD; 21.3 ± 2.11 years) with at least 5 years of soccer heading experience were included in the final analysis. Participants performed 10 soccer headers with soccer balls projected at a speed of 30 mph. Parameters related to blink reflex, including blink latency, differential latency, number of oscillations, delta 30, and excursions, were assessed by the EyeStat device at pre-heading baseline, and 2-h and 24-h post-heading.

**Results:**

Significant declines in blink reflex parameters were observed at specific post-heading timepoints compared to baseline. At 24-h post-heading, significant reductions were detected in the overall blink latency (*p* = 0.0255), the blink latencies of the right eye (*p* = 0.0411), ipsilateral latency (*p* = 0.0314) and contralateral latency (*p* = 0.0434). At 2-h post-heading, significant declines were observed in the overall delta 30 value (*p* = 0.0053) and delta 30 of the right eye (*p* = 0.0260). Both delta 30 values returned to baseline by the 24-h post-heading timepoint. No significant changes in the differential latency, number of oscillations, and excursion of the eye were found.

**Discussion:**

These findings suggest changes in the latency and delta 30 of a blink reflex is a viable measure of detection for neurological changes when monitoring subconcussive head impacts.

## Introduction

The need for early detection and preventative measures for concussions has been a focal point in public health discussions in the past two decades. Concussions can trigger acute and chronic impairments across diverse aspects of neurological function, encompassing cognition and sensorimotor control (1). Previous studies have substantiated an association between repetitive concussions and heightened risks for developing neurological abnormalities including neurodegenerative conditions like chronic traumatic encephalopathy (CTE) (2–5). Adding to this complexity, emerging evidence suggests that repetitive exposure to subconcussive head impacts that do not induce overt concussion-like symptoms may still result in similar neurological outcomes as observed in concussion cases (2, 6–14). While research in subconcussive injury has been undertaken for the past decade, mechanism of injury, diagnostic methods, and threshold at which subconcussive head impacts become injurious remain largely unknown.

The current understanding is that subconcussive head impacts, if sustained repetitively, can cause acute elevations in brain injury blood biomarkers, axonal microstructural injuries, and balance defects (13, 15–19). With chronicity, these neurological impairments may become irreversible and increase risks for developing early onset of neurodegenerative conditions (20, 21), including Alzheimer’s disease and CTE (5, 22). In the pursuit of establishing precise and sensitive objective measures for assessing brain health, emerging data suggest that the oculomotor system can serve as a valuable indicator of subtle changes caused by subconcussive head impacts (1, 9, 12, 13, 23, 24). For example, in a randomized controlled trial by using a controlled soccer heading model, Nowak et al. (10) demonstrated that 10 soccer headings induced acute impairments in binocular convergence and smooth pursuit in adult soccer players. These findings were corroborated in the context of American football, where players consistently exposed to subconcussive head impacts displayed deficits in binocular convergence for several weeks (12, 24) to until the post-season (13).

While these oculomotor tests provide insights in clinical practice, their performance can be influenced by subjective factors, underscoring the necessity for purely objective measures capable of capturing the subtle microtrauma caused by repetitive subconcussive head impacts. Addressing this need, the EyeStat was developed and is FDA approved to provide an objective assessment of the blink reflex (25). This head-set device administers a randomized puff of CO_2_ air to the sides of the eyes to trigger bilateral blink reflex. The induced corneal blink reflex objectively assesses the neuronal network loop involving trigeminal sensory nerves and the facial motor nerve innervation of the orbicularis oculi muscles (26). The EyeStat device installed with a highly sensitive internal camera collects measurements of blink latency, differential latency, number of oscillations, delta 30, and excursions. However, evidence regarding the device’s capability to detect changes induced by subconcussive head impacts is currently lacking.

Therefore, this proof-of-concept study aimed to investigate whether, and to what extent, the acute neurological effects of subconcussive head impacts manifest in the corneal blink reflex. We used our established controlled soccer heading model (27) to induce standardize head impacts while serially collecting blink reflex parameters at pre-heading baseline, 2-h, and 24-h post-heading. Our working hypothesis is that 10 bouts of soccer heading will trigger significant alterations in the blink latency, the number of oscillations, and excursions.

## Materials and methods

### Participants

**Figure 1:**
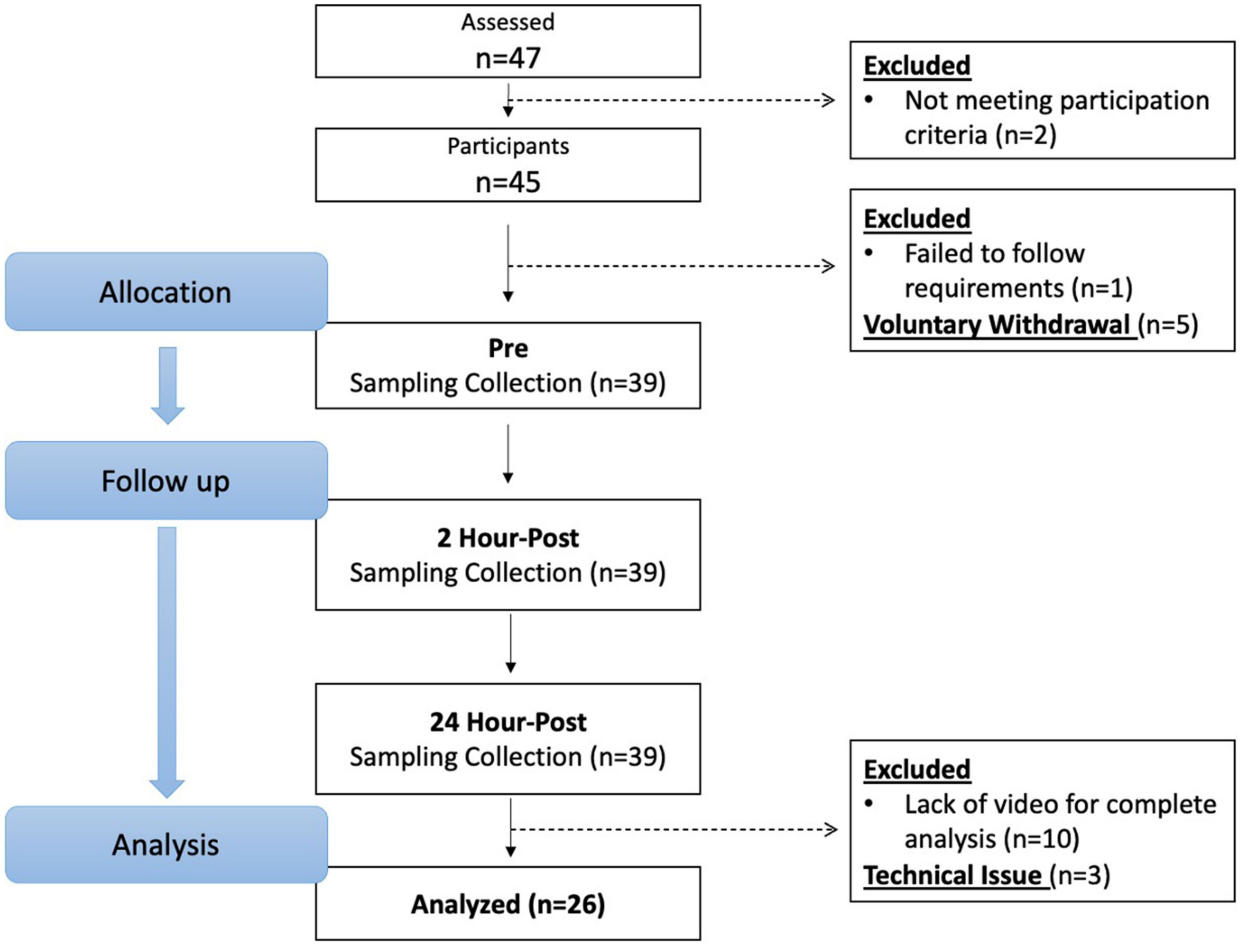
Participants flow chart.

From April 2021 through March 2022, 47 potential participants were screened, and 26 participants (15 females, 11 males: age ± SD; 21.3 ± 2.11 years) completed and were included in the study analysis (Figure 1). Potential participants were recruited by emails to university’s listserv, Indiana University Classified posting, and flyers posted on university and local boards. All potential participants filled out a health questionnaire that examined his/her current physical and mental health statuses, sport participation history, and history of concussion. Participants were asked to recall and self-report their history of concussion. This retrospective recall method has potential to be influenced by recall bias; however, it is a commonly used method and has been implemented in our previous studies (10, 15). The inclusion criteria for this project consisted of being between the ages of 18 and 26 years and having at least 5 years of soccer heading experience, a threshold chosen based on safety considerations to ensure sufficient skill and familiarity with proper heading techniques. Exclusion criteria was a history of head injury including concussion with symptoms remaining within 3 months prior to the study. Following the reports, all participants underwent a semi-structured diagnostic interview to assess their mental disorder diagnosis status. Participants who scored below the respective Diagnostic and Statistical Manual of Mental Disorders 5th Edition (DSM-5) thresholds and reported no current or prior history of major mental or learning disorders (e.g., attention-deficit/hyperactivity disorder, autism spectrum disorder, dyslexia) continued to the study participation. These conditions were excluded due to prior evidence (28, 29) suggesting that neurodevelopmental and learning disorders may be associated with altered oculomotor function and ophthalmologic deficits, which could confound blink reflex measurements and bias interpretation of subconcussive effects. Participants were blinded to the study aims and hypotheses. Sample sizes were determined by an *a priori* power analysis based on findings from our preliminary study (30), where we estimated a moderate effect size (Cohen’s d = 0.5) to detect subconcussive effects on blink reflex parameters. With an alpha level set at 0.05, 25 participants will yield at least 80% power to detect subconcussive effects.

### Study design

This study consisted of college-aged soccer players. Each participant underwent the blink reflex assessment at 3 time points: [pre-heading baseline, 2-h post-heading, and 24-h post-heading]. The order of sessions was fixed across all participants to allow for consistent within-subject comparisons and reduce variability. Participants performed 10 soccer headers and remained in the laboratory until the 2-h post-heading time point without engaging in strenuous cognitive or physical activities and returned to the laboratory 24 h later for the final data collection. This design was informed by our prior work demonstrating that visual and neuro-ophthalmologic function is impaired at 2 and 24 h following 10 soccer headers (9, 10). This approach enhances ecological validity by aligning with real-world field conditions, where immediate post-impact assessments are often impractical, whereas assessments at 2 and 24 h are more feasible for athletic trainers and team clinicians. All individuals were instructed to refrain from activities that may induce subconcussive head impacts, as well as alcohol and recreational drug use beginning 3 days prior to the start of the study and continuing until completing the 24-h post-intervention. The Indiana University Institutional Review Board approved the study protocol, which was also registered under ClinicalTrials.gov (NCT04880304).

### Subconcussion intervention

To induced controlled subconcussive head impacts, participants were tasked with executing 10 soccer headers, separated by a 1-min interval of rest between each header (10, 15, 27, 28, 31). A JUGS soccer machine (JPS Sports, Tualatin, Oregon) was used to launch a size 5 soccer ball that traveled approximately at 30 mph (13.4 m/s), aligning with the speed typically associated with balls kicked by adult soccer players on the slower-scale end (32). Participants stood approximately 40 feet away from the JUGS soccer machine and were instructed to head the ball in the air to a researcher standing 20 feet in front of the participant. This protocol was designed to replicate technique and intensity of real-world heading (33, 34). Soccer players experience head impacts ranging from approximately 20 g–100 g, with higher magnitude impacts occurring from goalkeeper’s punts and goal-kicks (33, 35, 36). To prioritize participant safety and comfort, we aimed to replicate impacts at a lower magnitude which are more typical of controlled drills and practice settings (35). Head impact kinematics were measured using a G-force triaxial accelerometer (GFroceTracker Inc., Ontario, CA) that was worn on participants’ heads and captured the linear and rotational head acceleration to quanitify the magnitude of each impacts.

### Blink reflex intervention

The EyeStat (BlinkCNS, Charleston, SC) device, approved by the FDA, provides an objective assessment of the blink reflex (25). The EyeStat has been validated to the gold-standard method of blink assessment in a laboratory setting (i.e., high speed videography and EMG) (37). The device consisted of a mask, stimulation system, camera, and a display screen. The device was held up on a tripod and participants were seated in front of the device. A trained researcher adjusted the participant and device so that their face was fully in the mask and their pupils were aligned with the green dotted line on the display screen. Each test started with a sample puff to allow participants to experience the sensation prior to recording. The entire test was roughly 30 s where 8 puffs of CO_2_ air was gently released from the right or left side of the mask. The puffs were randomized by order and by time in between puffs. During the test administration, the researcher was able to view and confirm that the participants eyes remained in alignment. The EyeStat device records latency, differential latency, oscillation, Delta-30, and lid excursion from both left and right eyes, as well as latency in ipsilateral and contralateral eyes based on CO_2_ air stimulus. The output parameters derived from the test recording and analysis are presented in Table 1.

**Table 1:** BlinkTBI output parameters.

Blink parameter	Units	Measured data
Blink latency	msecs	The time difference between the stimulation of the eye and the eye movement
Differential latency	msecs	The time difference between the beginning of the ipsilateral (same side as puff) eye movement and the beginning of the contralateral (opposite side as puff) eye movement
Number of oscillation	Total #	The frequency of up and down cycles of the upper eyelid movement after a blink is stimulated
Delta 30	msecs	The time difference between the ipsilateral (same side as puff) eye and the contralateral (opposite side as puff) eye movement after the eyelids have moved 30 pixels from their starting position
Lid excursion	Pixels	The distance the eyelid moves from the normal open lid position to closed position

### Statistical analysis

A series of repeated measures ANOVAs were conducted to examine the effect of time (pre-heading baseline, 2-h post-heading, 24-h post-heading) on each blink reflex outcome. Prior to each analysis, Mauchly’s Test of sphericity was performed. If a significant main effect of time was observed using ANOVA, Dunnett’s *post-hoc* tests were then used to compare baseline values against both the 2-h and 24-h post-heading time points to determine when the changes in blink reflex outcomes occurred (pre vs. 2-h; pre vs. 24-h). This approach was selected to reduce the risk of type I error from multiple comparisons while testing the hypotheses of post-heading changes. All analyses were performed using SPSS statistics version 25, with the level of statistical significance was set at *p* < 0.05.

## Results

### Demographics

Forty-seven individuals were assessed for eligibility, and 45 individuals who met the inclusion criteria and were free of exclusion criteria proceeded to the study. One individual was excluded due to not following participation requirements, and five individuals voluntarily withdrew before the pre-heading baseline time point due to lost interest or schedule conflict. As a result, 39 participants completed the protocol. The video files of 10 participants were corrupted during the uploading phase and three participants data was unable to be collected due to technical issues; therefore, their data was excluded from analysis and 26 participants were valid for analysis (Figure 1). Although our power analysis indicated that 25 participants would provide 80% power to detect moderate effects, the data loss from dropouts and technical issues may reduce statistical power for detecting smaller effects and limits generalizability to broader populations. Demographics and head impact kinematics are detailed in Table 2.

**Table 2:** Demographics and head impact kinematics.

Variables	Participants
*n*	26
Sex	11 M 15 F
Age, y	21.3 ± 2.11
BMI, kg/m^2^	24.13 ± 0.64
No. of previous concussion	0.23 ± 0.13
Soccer heading experience, y	9.46 ± 0.66
Race, *n*	
White	22
Black/African American	1
Asian	1
Indian	0
Hawaiian	0
Multiracial	2
Ethnicity, *n* (%)	
Not Latino/Hispanic	23
Latino/Hispanic	3
Head impact kinematics, median (IQR)^a^	
PLA, g	14.29 (11.25–19.28)
PRA, krad/s^2^	841.39 (580.28–1410.34)

All data is reported as means of each demographic ± standard deviations, unless otherwise specified. BMI, body mass index. IQR, interquartile range. PLA, peak linear acceleration. PRA, peak rotational acceleration. Krad, kiloradian.

a Median impact per header.

### Alterations in blink reflex after 10 headings

**Figure 2:**
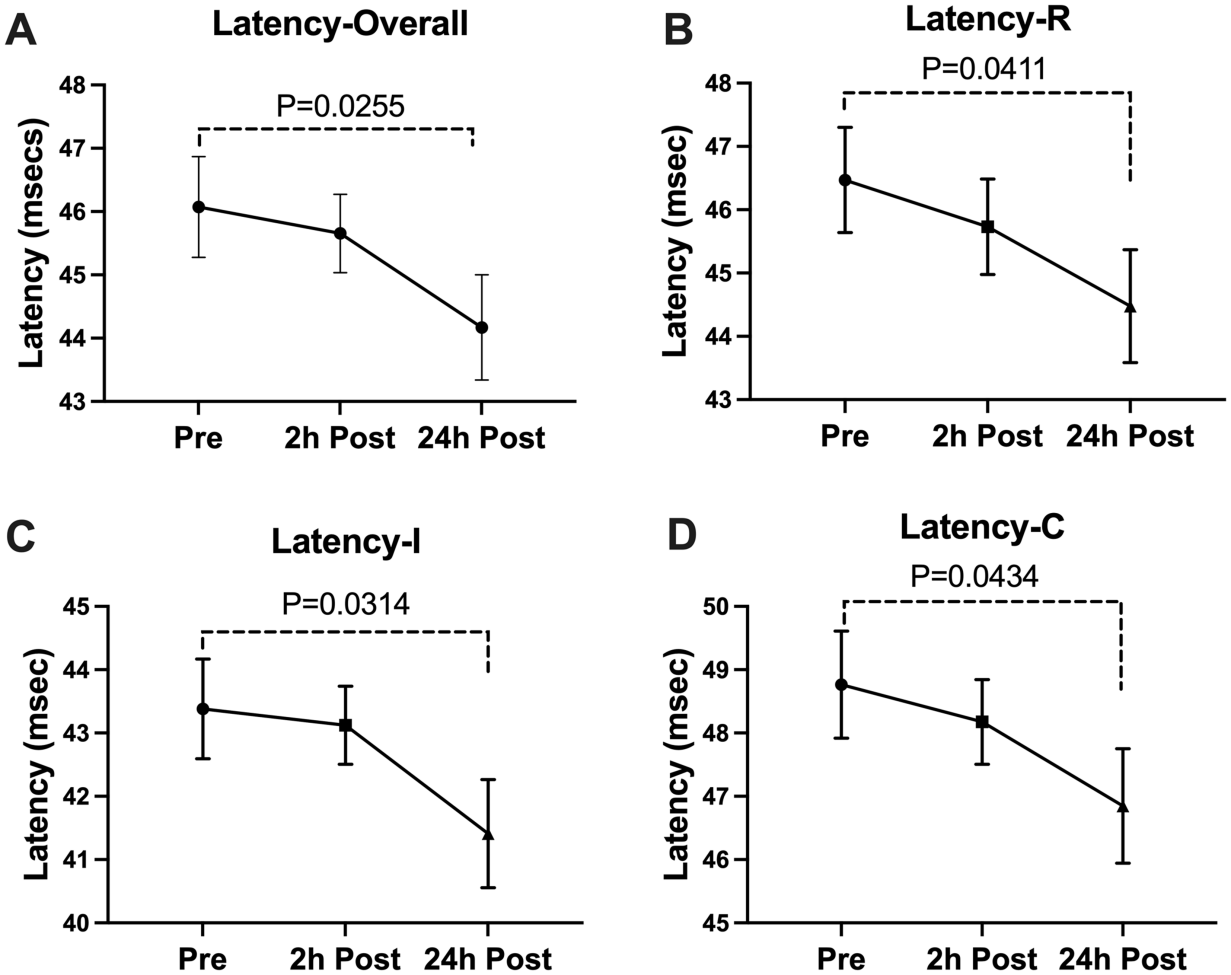
Subconcussive head impact effects on **(A)**overall latency, **(B)**right eye latency, **(C)** ipsilateral latency, and (D) contralateral latency. Blink latency metrics were measured prior to the 10 soccer headings and twice after at the 2-hour time point and the 24-hour time point. At 24-hours after the soccer heading protocol a significant decrease was observed in the overall latency (Latency-Overall, *p* = 0.0255), the latency in the right eye (Latency-R, *p* = 0.0411), the ipsilateral latency (Latency-I, *p* = 0.0314), and the contralateral latency (Latency-C, *p* = 0.0434). Values graphed represent means at each time point and the standard error of the mean (SEM).

**Figure 3:**
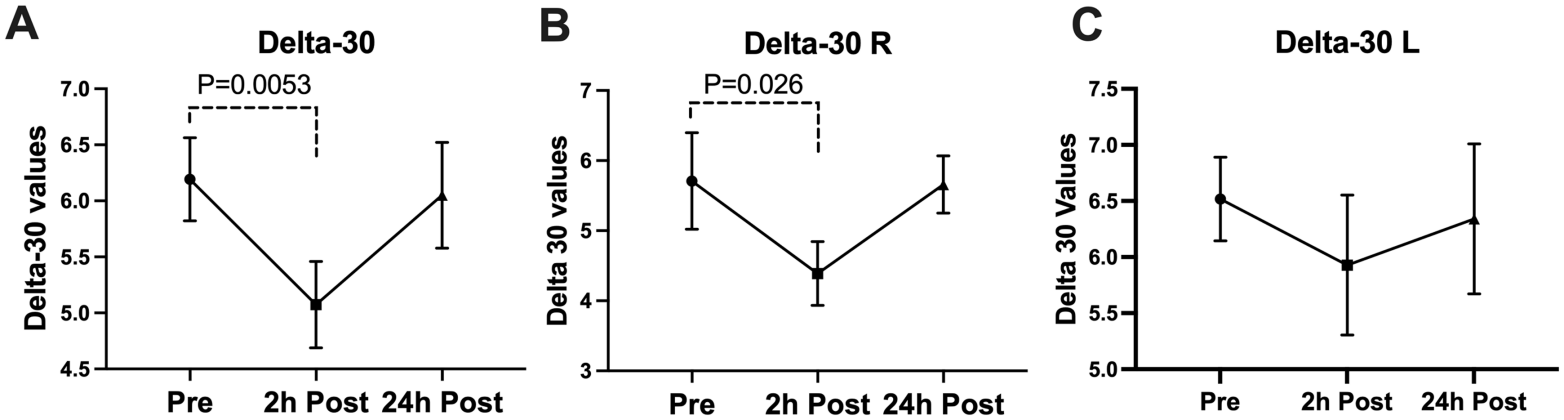
Subconcussive head impact effects on the **(A)** overall delta 30 value, **(B)** right eye delta 30 value, and **(C)** left eye delta 30 value. Blink latency was measured prior to the 10 soccer headings and twice after at the 2-hour time point and the 24-hour time point. At 2-hours after the soccer heading a significant decrease was observed in the overall delta 30 value (delta 30-Overall, *p* = 0.0053) and the delta 30 of the right eye (delta 30-R, *p* = 0.0260). The values returned to their baseline ranges at 24-hours post soccer heading. Values graphed represent means at each time point and the standard error of the mean (SEM).

**Figure 4:**
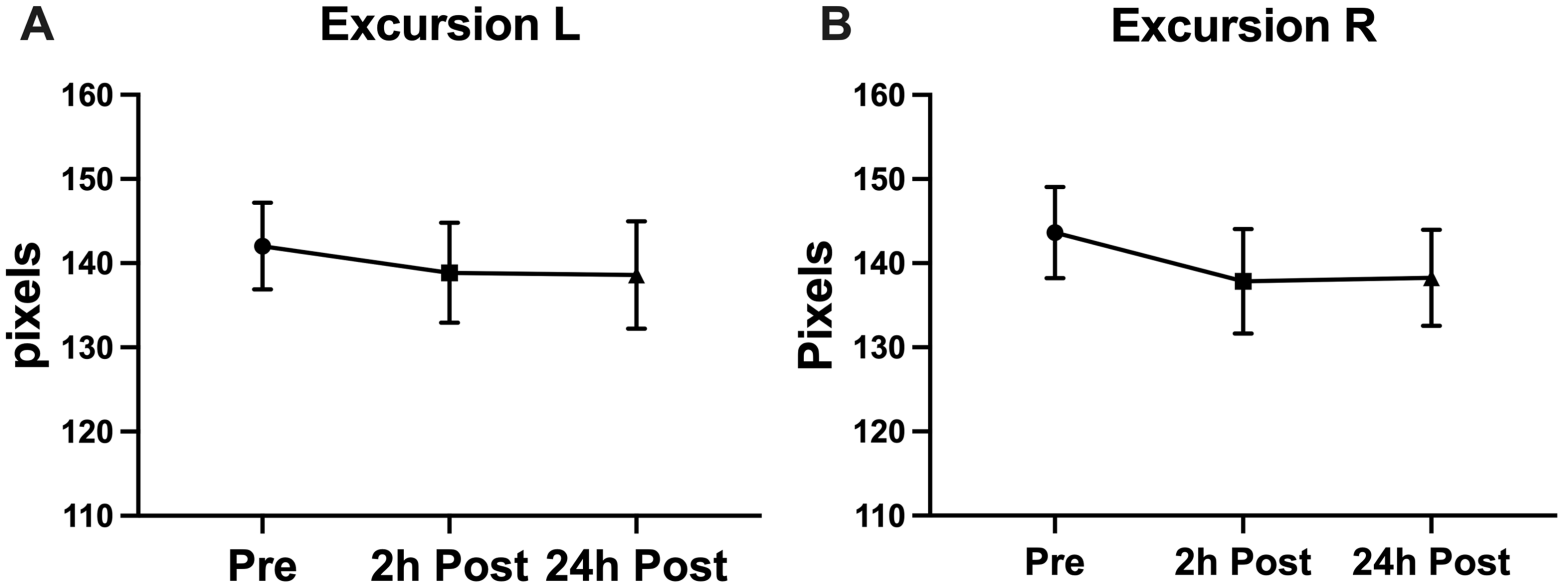
Subconcussive head impact effects on **(A)** left eye excursion and **(B)** right eye excursion. Despite expecting to see a change in the excursion of the blink during the blink reflex, a significant change in the blink excursion was not observed. Values graphed represent means at each time point and the standard error of the mean (SEM).

A significant decline was seen in the blink latency of participants at 24-h post-heading. This decline was seen in the overall latency of the eye, the latency in the right eye, the ipsilateral latency, and the contralateral latency of the eye [Latency-Overall 95%CI (0.224, 3.579), *p* = 0.0255; Latency-R 95%CI (0.076, 3.912), *p* = 0.0411; Latency-I 95%CI (0.167, 3.775), *p* = 0.0314; Latency-C 95%CI (0.053, 3.778), *p* = 0.0434] (Figure 2). There was a significant decrease in the overall delta 30 value and the right eye’s delta 30 value at 2-h post-heading compared to the baseline [delta 30-Overall 95%CI (0.330, 1.910), *p* = 0.0053; delta 30-R 95%CI (0.152, 2.486), *p* = 0.0260]. By 24-h post-heading, both values had returned to levels comparable to baseline, with no significant differences observed [delta 30-Overall 95%CI (−0.854, 1.139), *p* = 0.9206; delta 30-R 95%CI (−1.161, 1.262), *p* = 0.9927] (Figure 3). No significant changes in the differential latency, number of oscillations, or excursion of the eye were observed (Figure 4). Detailed outputs are summarized in Table 3.

**Table 3:** Subconcussive effects on blink reflex parameters.

Variable	Pre	2-hour post	*p*-value(2h vs. pre)	24-hour post	*p*-value(24h vs. Pre)
Latency (msecs)					
Latency-Overall	46.1 ± 4.1	45.7 ± 3.2	0.6991	44.2 ± 4.0	0.0255*
Latency-L	45.7 ± 4.4	45.6 ± 2.8	0.9851	43.8 ± 4.8	0.1190
Latency-R	46.5 ± 4.2	45.7 ± 3.8	0.4113	44.5 ± 4.3	0.0411*
Latency-I	43.4 ± 4.0	43.1 ± 3.2	0.8784	41.4 ± 4.1	0.0314*
Latency-C	48.8 ± 4.3	48.2 ± 3.4	0.5300	46.8 ± 4.3	0.0434*
Differential Latency	5.4 ± 1.9	5.2 ± 1.8	0.7760	5.9 ± 2.6	0.4704
Delta 30 (msecs)					
Delta 30-Overall	6.2 ± 1.9	5.1 ± 1.9	0.0053*	6.1 ± 2.3	0.9206
Delta 30-L	6.5 ± 1.9	5.9 ± 3.0	0.4916	6.3 ± 3.1	0.9413
Delta 30-R	5.7 ± 3.2	4.4 ± 2.2	0.0260*	5.7 ± 1.7	0.9927
Oscillation (total #)					
Oscillation-Overall	11.7 ± 10.3	14.3 ± 12.4	0.5111	11.9 ± 6.2	0.9739
Excursion (pixels)					
Excursion-Overall	142.8 ± 26.5	138.3 ± 30.4	0.2138	138.4 ± 26.3	0.1953
Excursion-L	142.0 ± 26.2	138.9 ± 30.3	0.5087	138.6 ± 30.5	0.4399
Excursion-R	143.6 ± 27.6	137.9 ± 31.6	0.0909	138.3 ± 27.3	0.1180

* indicates statistically significant changes relative to pre-heading baseline. Values are expressed as mean ± standard deviation. Latency and delta 30 are expressed in milliseconds. Oscillation is expressed as total numbers. Excursion is expressed in pixels. -L, Left eye; -R, Right eye; -I, Ipsilateral eye; -C, Contralateral eye.

## Discussion

This study aimed to evaluate the utility of air puff–induced blink reflex metrics in detecting neurological alterations following subconcussive head impacts. Key findings include a significant reduction in blink latency observed 24 h after participants completed 10 controlled soccer headers, along with a decrease in delta 30 values at 2 h post-heading. In contrast, no significant changes were detected in differential latency, number of oscillations, or eye excursion. These results suggest that blink latency and delta 30 may serve as sensitive markers for subtle neurological changes associated with subconcussive head impacts (Figure 4).

Our findings demonstrate a decrease in blink latency following the subconcussion intervention, indicating that soccer heading may induce acute neurological alterations reflected in blink reflex metrics. Repeated head impacts have been associated with impaired release of gamma aminobutyric acid (GABA), a neurotransmitter that plays a role in modulating dopamine release (7). Changes in dopamine occur with acute traumatic brain injury (38), with dopamine known to influence NMDA receptor activity. Notably, reductions in NMDA receptor subunits (NR1, NR2A, NR2B) have been reported 6–12 h post-injury in rodent models (39). These dopaminergic disruptions may partly account for the acute blink latency changes observed in the present study. Additionally, the observed decrease in blink latency, potentially linked to reduced dopamine levels, aligns with findings from various neurological disorders characterized by cognitive deficits (40). Prior research has also reported decreased blink latency following damage to multiple regions of the cerebral cortex (19). Although the precise mechanisms underlying the decline in latency following subconcussive impacts remain unclear, these findings highlight the potential of blink latency as a sensitive indicator of neurological response to repetitive head impacts. Likewise, a notable decrease in delta 30 was observed 2-h after the soccer headings. As a relatively novel parameter in blink reflex analysis, the decrease in delta 30 may suggest heightened neural excitability induced by subconcussive impacts, contributing to the temporal decline of the blink reflex. Further investigation is warranted to comprehensively grasp the influence of subconcussive head impacts on blink reflex and its potential utility in discerning neurological effects of subconcussive head impacts.

Comparative analysis of blink reflex data from this study with findings from concussion-based research reveals notable differences. Although our study focused on subconcussive head impacts, prior research examining blink reflex responses within 48 h post-concussion in football players reported significant reductions in latency, differential latency, time to close, time to open, and number of oscillations, but no changes in delta 30 (41). These discrepancies may reflect fundamental differences in the pathophysiology and injury mechanisms between concussion and subconcussion. No change in differential latency in the present study of soccer headings may suggest preserved bilateral blink reflex synchrony, indicating that subcortical coordination pathways remain intact at this stage of subconcussive exposure. Thus, our hypothesis was not supported as it relates to significant changes in differential latency, number of oscillations and lid excursion post head impacts. Our initial hypothesis proposed an increase in these values, given the anticipated state of hyperexcitability after head impacts. Subsequent to repetitive head impacts, subtle neural changes have been observed, such as an elevation in central autonomic network connectivity (42). These observations align with the anticipated consequences of brain damage. It is plausible that either the neurological changes after headings were not substantial enough or occurring with the acute time frame in which blink reflex was measured to induce noticeable alterations in the neural pathways governing these reflexes, or these specific variables may not serve as reliable indicators to gage the effects of subconcussive head impacts. Further investigation is imperative to elucidate the underlying reasons for the observed results.

Caution is advised in interpreting the results of this study due to potential limitations. Although participants were monitored between the soccer headings and 2-h post-heading time point, the same level of rigorous monitoring did not take place between 2-h and 24-h data collection. This may have led to additional factors influencing the outcome of the data. Environmental factors such as lighting and sound in the room may have impacted the blink reflex, although all participants at all time points took place in the identical environment. Although the latency reductions were statistically significant, the magnitude of change may not meet thresholds of clinical impairment. Future work is needed to establish normative ranges and minimal clinically important differences for these measures. Lastly, the findings of this study should be interpreted with caution. Specifically, the sample size was limited and drawn from a single site, restricting generalizability to the broader population. Additionally, the observed large standard deviations may obscure subtle effects. Future studies should increase sample size and head impact dosage to delineate the relationships between subconcussive head impacts and blink reflex.

## Conclusion

This study contributes initial evidence supporting the partial capacity of the blink reflex to gage certain neurological changes induced by acute subconcussive head impacts. Specifically, alterations in blink latency and delta 30 of the blink reflex were identified as reflective indicators of subconcussive neural stress. Future studies may benefit from delving deeper into the underlying mechanisms by investigating potential impairments of the neural pathway of the blink reflex following subconcussive head impacts. Such explorations hold promise in advancing our understanding of the intricate effects of subconcussive head impacts on neurological function.

## Data Availability

The raw data supporting the conclusions of this article will be made available by the authors, without undue reservation.
